# A Multimodal Approach in the Treatment of Persistent Post-COVID

**DOI:** 10.3390/diseases10040097

**Published:** 2022-11-01

**Authors:** Tobias Romeyke

**Affiliations:** 1 Medical Informatics and Technology, Institute for Management and Economics in Health Care, UMIT—University of Health Sciences, 6060 Hall in Tirol, Austria; tobias.romeyke@umit-tirol.at; 2 Waldhausklinik Deuringen, Acute Hospital for Internal Medicine, Pain Therapy, Complementary and Individualized Patient Centered Medicine, 86391 Deuringen, Germany

**Keywords:** post-COVID, Long-COVID, COVID-19, complementary medicine, whole-body hyperthermia, treatment, multimodal approach, inflammation

## Abstract

Background: Many patients suffer from the consequences of a COVID infection. The so-called long or post-COVID syndrome affects the quality of life of patients and can lead to severe physical impairments. There are currently no suitable therapies for the treatment of long/post-COVID. Case presentation: A 49-year-old patient with post-COVID was admitted to a specialized clinic to carry out a multimodal therapy approach in the event of a therapy-resistant course. In addition to pronounced fatigue, sleep disorders, inner restlessness, and depression were seen in the patients’ high levels of suffering. A naturopathic complex therapy including systemic whole-body hyperthermia was carried out. Well-being and physical well-being were recorded using the visual analog scale, and depression was recorded using the Patient Health Questionnaire Depression (PHQ-D). There was close monitoring of the vital parameters, and an evaluation of the therapy result was performed. Discussion and Conclusion: The implementation of a naturopathic complex therapy including systemic whole-body hyperthermia was able to significantly improve the mental state, physical well-being, and mood of the patient. Since there are still no evidence-based therapy recommendations for the treatment of long/post-COVID, clinical research is called upon to intensively deal with this topic and to examine treatment concepts.

## 1. Introduction

### 1.1. Post-COVID

Persistent symptoms four weeks after acute COVID-19 illness are known as post-acute COVID syndromes. Persistent or emerging symptoms can be seen after 12 weeks with post-COVID syndromes, and both entities together are known as long-COVID [1,2].

The exact causes are not yet known. It can be assumed, without scientific evidence, that post-COVID syndrome could be associated with chronic subclinical systemic inflammation.

Initial studies indicate that the frequency of post-COVID syndrome can be assumed to be up to 15%, depending on the patient population examined [3]. Currently, no causal relationship between the frequency and pre-existing comorbidities is seen [4].

The affected patients report fatigue, muscle stiffness, and sleep disorders 6 months after an inpatient stay [5]. One of the current assumptions is an immunological disorder, accompanied by acute inflammatory processes and the release of inflammatory mediators that negatively influence post-COVID symptoms [6].

The aim of therapy should be to alleviate symptoms and avoid chronification. Complex clinical pictures such as long/post-COVID require an interdisciplinary approach with a view to the whole person and continuity in care [7].

This includes shared decision making and a holistic assessment to identify and comprehensively treat patient complaints [2]. Multidisciplinary clinics with specialized staff are moving into focus when it comes to caring for those affected [8].

Data on possible treatment options for post-COVID are rare, so this case study will present a multimodal therapeutic approach using conventional therapies, complementary medicine, and systemic whole-body hyperthermia.

### 1.2. Complementary Medicine

From a global perspective, the demand for complementary therapies is unbroken [9].

The integration of complementary therapy methods in conventional medicine is now practiced and further developed in many health systems worldwide [10].

Complementary and alternative therapy methods are also used for the supplementary treatment of COVID-19, and their effects are evaluated [11].

The consequences of a COVID-19 infection with the search for suitable treatment strategies also pose major challenges for those involved in healthcare worldwide.

In a scoping review by Kim et al. 2022, scientific work (review, RCT, observational, study, case report) about complementary medicine and post-COVID was evaluated [12].

Two clinical studies, a prospective case–control study and a case study, suggest that herbal medications may be effective in relieving the symptoms of lung dysfunction [12,13,14].

However, common symptoms such as tiredness or fatigue are not evaluated in the studies, and there is still little evidence of the effectiveness of the methods used.

Therefore, the need for further investigation and scientific debate is of great importance. The use of complementary procedures in addition to conventional therapy is described and evaluated in this study. A complex therapy (operation and procedure code 8-975) is used, which is already established in the German healthcare system and can serve as a model for other healthcare systems [15]. Systemic whole-body hyperthermia is integrated into this complex therapy as heat therapy.

### 1.3. Whole-Body Hyperthermia

At a body temperature of 37.0–37.5 °C, the physiological and cellular processes work optimally for humans. In the case of infections, the human body reacts with fever, i.e., its body temperature rises to activate defense mechanisms. The physically generated increase in body temperature is called passive hyperthermia. Hyperthermia has an immune-stimulating or modulating effect [16]. Hyperthermia is also intended to improve the supply of nutrients to the cells. There is an increase in the intra- and extracellular regeneration processes. Whole-body hyperthermia is also called “systemic hyperthermia”, which involves the entire organism. Their effect is described as immunostimulating and modulating and can promote various physiological functions of immunocompetent cells [17]. Analyses of the use of artificially generated thermotherapeutic interventions showed an initially falling cortisol level, which rose again with increasing heat disposition [18].

Cortisol, also known as hydrocortisone, is an endogenous hormone that is produced in the adrenal cortex. It is one of the glucocorticoids. Cortisol affects blood sugar and fat metabolism, delays water excretion, and has an anti-inflammatory effect.

There are various medical–therapeutic application methods, for example, infrared-A whole-body hyperthermia, in which infrared-A radiation is used to increase body temperature through water filtration. In the present study, indirect reflection-controlled infrared radiation (Heckel) was used. There is already an increasing number of scientific studies on various clinical pictures [19,20,21].

The results of scientific studies on the use of systemic whole-body hyperthermia in long/post-COVID have not yet been published.

## 2. Case

A 49-year-old patient was admitted for inpatient treatment with the post-COVID syndrome and postviral chronic fatigue syndrome. The SARS-CoV-2 infection was 12 months ago. The patient was not hospitalized. She returned to work after three weeks, although she still did not feel well.

The patient reported severe sleep disorders, weight gain, and depression since the COVID infection. Headaches and pain in the joints occurred again and again at short intervals. An inflammatory rheumatic disease was ruled out.

The patient constantly felt weak and tired, had a pronounced feeling of weakness, and was listless. She gained weight due to water retention. Any physical exertion led to a worsening of the symptoms and to a pronounced feeling of exhaustion, which then lasted for several days. Sleep was disturbed, she did not have a fever, she had attacks of sweating at night, and she did not drink alcohol. She smoked nicotine until two years ago. The appetite was normal, and the diet was normal.

The patient lived alone and could no longer go about her everyday life normally. Above all, she could no longer perform sports, which greatly affected her. Her weight increased by 10 kg in the last year. The therapies carried out on an outpatient basis were not effective, which was why the patient was admitted to the hospital to carry out a multimodal therapy approach.

### 2.1. Physical Status at Admission

The patient experienced slowed consciousness and an ill general condition. Musculature was normal, and skin color and turgor were normal. The body mass index was 27.

Head/Neck: Spontaneous pressure or percussion pain in the calvaria. Nerve exit free. Pupils isocor medium, light reaction ipsilateral on both sides. Hearing unremarkable. Oral mucosa moist, no fetor.

External neck/thorax: Lymph nodes unremarkable. No stridor. Thyroid normal, shiftable when swallowing. No jugular vein congestion. No pathological flow noise over the carotids. Thorax normal. Axillary lymph nodes not enlarged. Breathing normal.

Pulmo: Sonorous percussion sound, vesicular breathing sound. No rattling noises.

Cor: Regular heartbeat. HR 78/min. RR right 129/91 mmHg. RR left 136/99 mmHg.

Abdomen: Abdominal wall soft. Liver dull and spleen not enlarged, palpable. Kidney position free on both sides. No resistances. No hernias. No lymph nodes. Intestinal noises downright. No pressure pain.

Spine: No percussion and pressure pain in the cervical, thoracic, and lumbar spine. No myogelosis. Lasegue negative.

Extremities: Mobility free. Varices on the left. No edema.

Neurology: Roughly neurologically unremarkable. No pathological reflexes. Cranial nerves, unremarkable. No meningism. No tremor. The patient was mentally clear, responsive, and well-oriented, but clearly depressed.

ECG: Sinus rhythm, position type normal, heart rate 75/min, and no acute repolarization disorders.

Results of the admission assessment:

The evaluation of the Patient Health Questionnaire Depression (PHQ-D) resulted in 11 points (suspected major depressive disorder, moderate severity) (Table 1).

The extent of the pain-related disabilities was recorded using the Pain Disability Index (PDI) and was 40.5 out of 70 points.

Disabilities mean “the extent to which chronic pain interferes with a person’s ability to engage in various life activities”.

These include, for example, self-sufficiency, work, sex life, social activities, recreation, family, and domestic obligations.

### 2.2. Implementation of a Naturopathic Complex Therapy with the Integration of Systemic Whole-Body Hyperthermia

**Figure 1:**
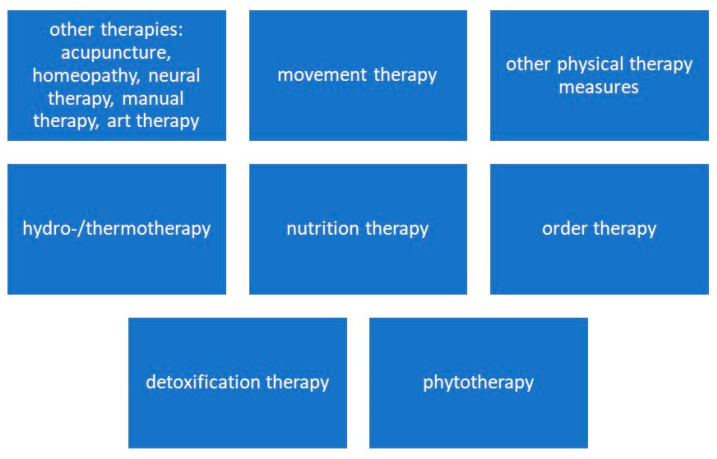
Therapy components of naturopathic complex therapy.

In view of the constellation of symptoms and the resistance to outpatient therapy, a naturopathic complex therapy was carried out in addition to conventional medicine. Figure 1 shows the therapy components of naturopathic complex therapy. This is laid down in the catalog of procedures for acute inpatient hospital care in Germany. The German procedure classification (operations and procedure key—OPS) is the official classification for the encoding of operations, procedures, and general medical measures. Naturopathic complex therapy can be carried out by specialized clinics in Germany.

As part of a multimodal, interdisciplinary therapy approach, therapy procedures from the areas of hydrotherapy/thermotherapy, regulatory therapy, other physical measures, movement therapy, draining procedures, and other therapies were carried out.

The physiotherapeutic and physical interventions (whole-body hyperthermia, foot reflex zone massage, lymphatic drainage, as well as other naturopathic therapy methods (liniment with Solum oil, ozone therapy)) were well-accepted by the patient and rated positively.

The focus was on thermotherapy using whole-body hyperthermia with the aim of stimulating the metabolism, deep muscular relaxation, vegetative stimulation, and immune stimulation.

A total of five hyperthermia applications were performed on the second, fourth, seventh, ninth, and eleventh days.

**Figure 2:**
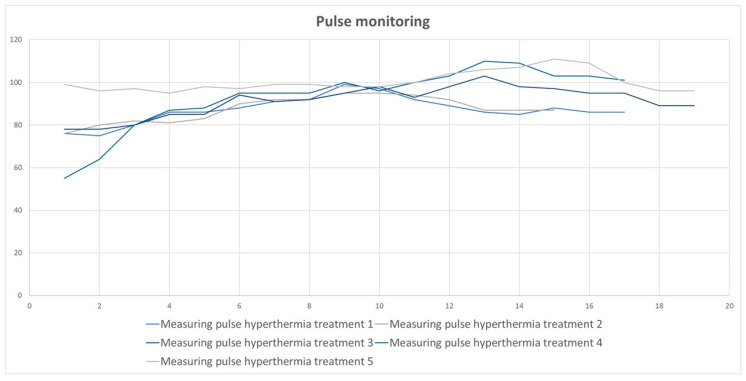
The results of pulse monitoring.

The heart rate was measured at five-minute intervals during hyperthermia treatment. The average heart rate during the five hyperthermia treatments was 92.15/min. In the fourth and fifth hyperthermia, the pulse was above the average (Figure 2).

**Figure 3:**
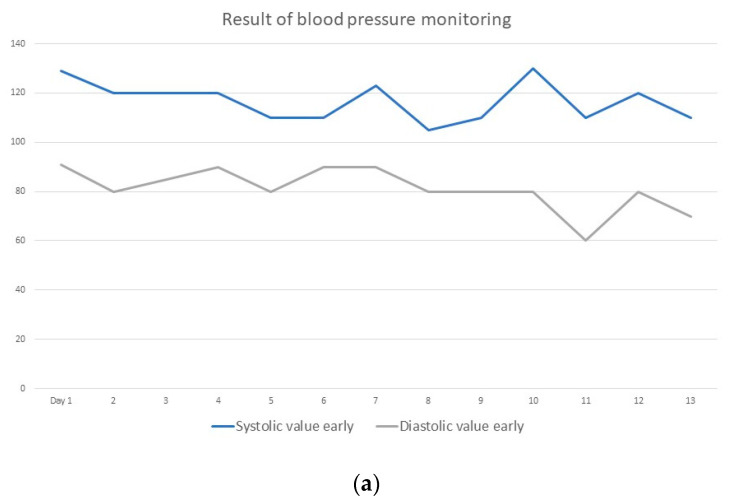

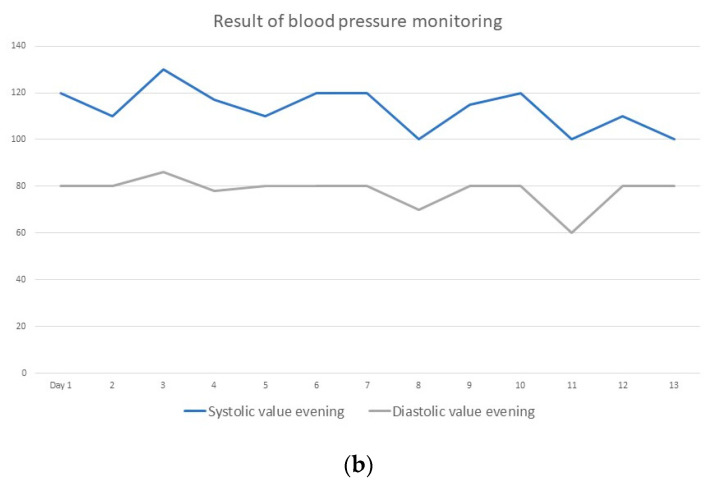
(**a**) Blood pressure monitoring in the morning; (**b**) blood pressure monitoring in the evening.

The blood pressure values were in the normal range (morning and evening) and during the application of hyperthermia at an average of 125/80 mm Hg (Figure 3a,b).

The average treatment time was 80.2 min.

The procedures were well-tolerated and resulted in a reduction in the intensity of the symptoms over the course of the inpatient stay.

Exercise therapy was used with the aim of strength conditioning and endurance, energetic stimulation, and improvement in cognition and coordination.

Further autogenic training took place for psycho-vegetative stabilization.

In addition, the patient received regulatory therapy. The focus here was on methods for dealing with the disease and coping strategies.

The patient was also looked after psychotherapeutically, and the pain therapist treated her with acupuncture.

Naturally, we also administered healing earth and the trace elements of selenium, zinc, and ginkgo.

Healing earth was administered to reduce heartburn and acid-related stomach pain.

All the procedures were well-tolerated by the patient and brought relief from the symptoms by the end of her inpatient stay.

The main aspects of the patient’s written questionnaire at the end of her stay in the hospital were expressed as follows:

- “I was included in the therapy as a whole person”;
- “The staff responded to my questions”;
- “Especially the hyperthermia, the liniments, relaxation-leading procedures, and the physiotherapeutic measures helped”;
- “My quality of life has improved significantly.”

The results of the discharge management showed a significant improvement in psychological well-being (PHQ-D 4 points), an improvement in mood (well-being) (VAS 2/10), and physical condition (3.0 out of 10).

Sleep disturbances improved from 7.1/10 at admission to 4.3/10 VAS at discharge.

## 3. Discussion

The pathogenesis of the post-COVID syndrome is unclear. Many factors may play a role, such as nervous system dysfunction, chronic (hyper)inflammation, and thromboembolism [22]. SARS-CoV-2 infection can contribute to fatigue.

The fatigue associated with long/post-COVID can be seen as a multisystem disease with the dysregulation of the immune system, the vascular system, and the nervous system, as well as cellular energy metabolism [23].

Studies show that even patients with milder courses without hospitalization can develop multiorganic disabilities and severe symptoms [24]. Persistent exhaustion, depression, unfounded fears, and sleep disorders affect the psychological well-being of post-COVID patients [25]. This affects patients’ quality of life and leads to an increase in work absenteeism.

Depression is also a frequent consequence of COVID-19 [26], which is why psychological diagnostics should be given high priority. The patient’s moderate depression in this case study could be treated without the use of psychotropic drugs and by means of psychotherapy, hyperthermia, and relaxation techniques [27]. The methods used show a possible influence on the mental state and underpin the therapy in a multimodal setting. Drug therapies that are evidence-based are currently not available, so the focus should be on patient-specific therapy based on the key symptoms of post-COVID patients.

Multimodal therapy concepts are designed to comprehensively treat the patient’s symptoms. This is achieved through a variety of therapies and the use of different specialist disciplines in the sense of a holistic, patient-centered therapy approach.

Linked to this is the therapy goal of avoiding increased intake of sedatives, opioids, and antidepressants, which is often described in post-COVID patients [28].

Since many therapy concepts for the treatment of post-COVID are currently being tested, they should be scientifically documented and evaluated. The aim is to establish effective and efficient therapies to be able to help the numerous affected patients in the long term and to reduce the burden of this disease.

## Figures and Tables

**Table 1:** Detailed answers on psychological well-being at the time of admission (Patient Health Questionnaire Depression).

Over the Past 2 Weeks, How Often Have You Felt Affected by the Following Complaints?	
- Little interest or pleasure in your work	Not at all
- Depression, sadness, or hopelessness	Not at all
- Difficulty falling asleep or staying asleep, or increased sleep	Almost every day
- Feeling tired or lacking in energy	Almost every day
- Decreased appetite or excessive need to eat	On some days
- Bad opinion of yourself; feeling a failure to be or to have disappointed the family	Not at all
- Difficulty concentrating on something, e.g., reading the newspaper or watching TV	Almost every day
- Were your movements or speech slowed down enough for others to notice? Or, on the contrary, were you “fidgety” or restless? Does this mean you have a stronger urge to move than usual?	On single days
- Thoughts that you would rather be dead or want to hurt yourself?	Not at all
In the last 4 weeks, have you had an anxiety attack (sudden feeling of fear or panic)?	No
Please indicate how much the problems described above made it difficult for you to do your job, take care of your household, or with other people to cope.	Very strong
Over the past 4 weeks, how often have you felt bothered by the following symptoms?	
Feeling nervous, anxious, tense, or overly concerned	Not at all
Feeling restless, making it difficult to sit still	Not at all
Easily fatigable	More than half the days
Muscle tightness, muscle pain	Not at all
Difficulty falling or staying asleep	More than half the days
Difficulty concentrating on something, such as reading or watching TV	More than half the days
Slight irritability, hypersensitivity	More than half the days

Impairments in mood, physical condition, and sleep were measured using the visual analog scale (VAS): VAS impairment in mood (well-being): VAS 7.9 out of 10, impairment in physical condition: VAS 8.0 out of 10 and sleep impairment: VAS 7.1 out of 10.

## Data Availability

Data are contained within the article.
